# Supplementary material for: Environmental Regulation of Toxin Production in Bacillus anthracis
Source: bioRxiv. 2025 Oct 7:2025.10.02.679957. Preprint. [Version 1] doi: 10.1101/2025.10.02.679957 (PMC12747272; doi:10.1101/2025.10.02.679957)
Supplement: Supplement 1 [file NIHPP2025.10.02.679957v1-supplement-1.pdf]

bioRxiv preprint doi: https://doi.org/10.1101/2025.10.02.679957; this version posted October 7, 2025. The copyright holder for this preprint (which was not certified by peer review) is the author/funder. This article is a US Government work. It is not subject to copyright under 17 USC 105 and is also made available for use under a CC0 license.

Supplementary figure 1.

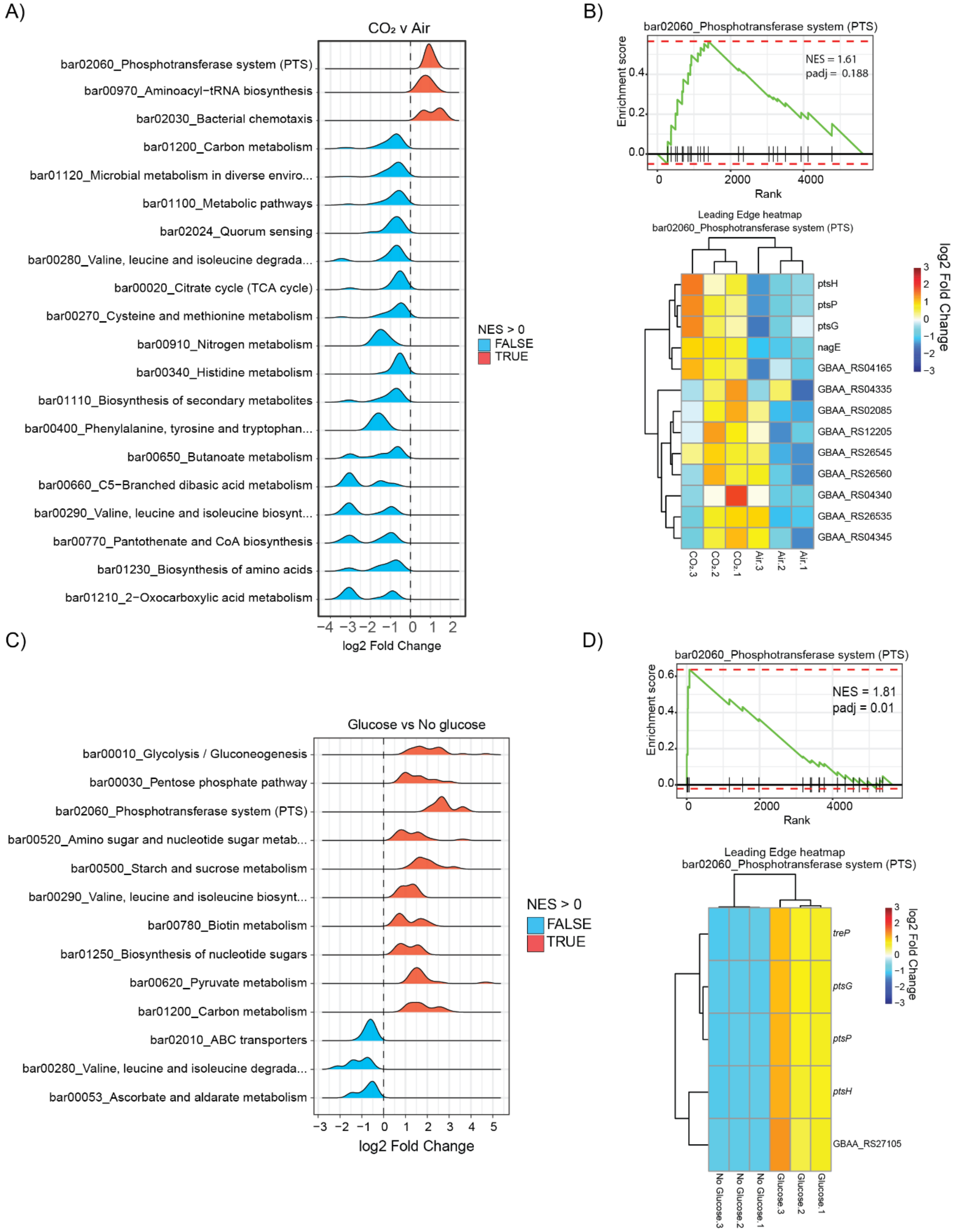

A) Ridge plots representing significantly dysregulated metabolic pathways in *B. anthracis* Wt grown under toxin-producing conditions (glucose + 5% CO_2) versus non-toxin-producing

bioRxiv preprint doi: https://doi.org/10.1101/2025.10.02.679957; this version posted October 7, 2025. The copyright holder for this preprint (which was not certified by peer review) is the author/funder. This article is a US Government work. It is not subject to copyright under 17 USC 105 and is also made available for use under a CC0 license.

conditions (glucose + air). Up-regulated pathways (NES > 0) are colored red, while down-regulated pathways (NES < 0) are colored blue.

B) Representative enrichment score for the PTS pathways (bar02060_PTS). Vertical bars represent the grouping pattern of individual genes of the PTS pathway based on their net enrichment scores. The heatmap below indicates the leading genes identified based on their enrichment scores.

C) Ridge plots representing significantly dysregulated metabolic pathways in *B. anthracis* Wt grown under toxin-producing conditions (glucose + 5% CO_2) versus non-toxin-producing conditions (no glucose + 5% CO_2). Up-regulated pathways (NES > 0) are colored red, while down-regulated pathways (NES < 0) are colored blue.

D) Representative enrichment score for the PTS pathways (bar02060_PTS). Vertical bars represent the grouping pattern of individual genes of the PTS pathway based on their net enrichment scores. The heatmap below indicates the leading genes identified based on their enrichment scores.

bioRxiv preprint doi: https://doi.org/10.1101/2025.10.02.679957; this version posted October 7, 2025. The copyright holder for this preprint (which was not certified by peer review) is the author/funder. This article is a US Government work. It is not subject to copyright under 17 USC 105 and is also made available for use under a CC0 license.

Supplementary figure 2.

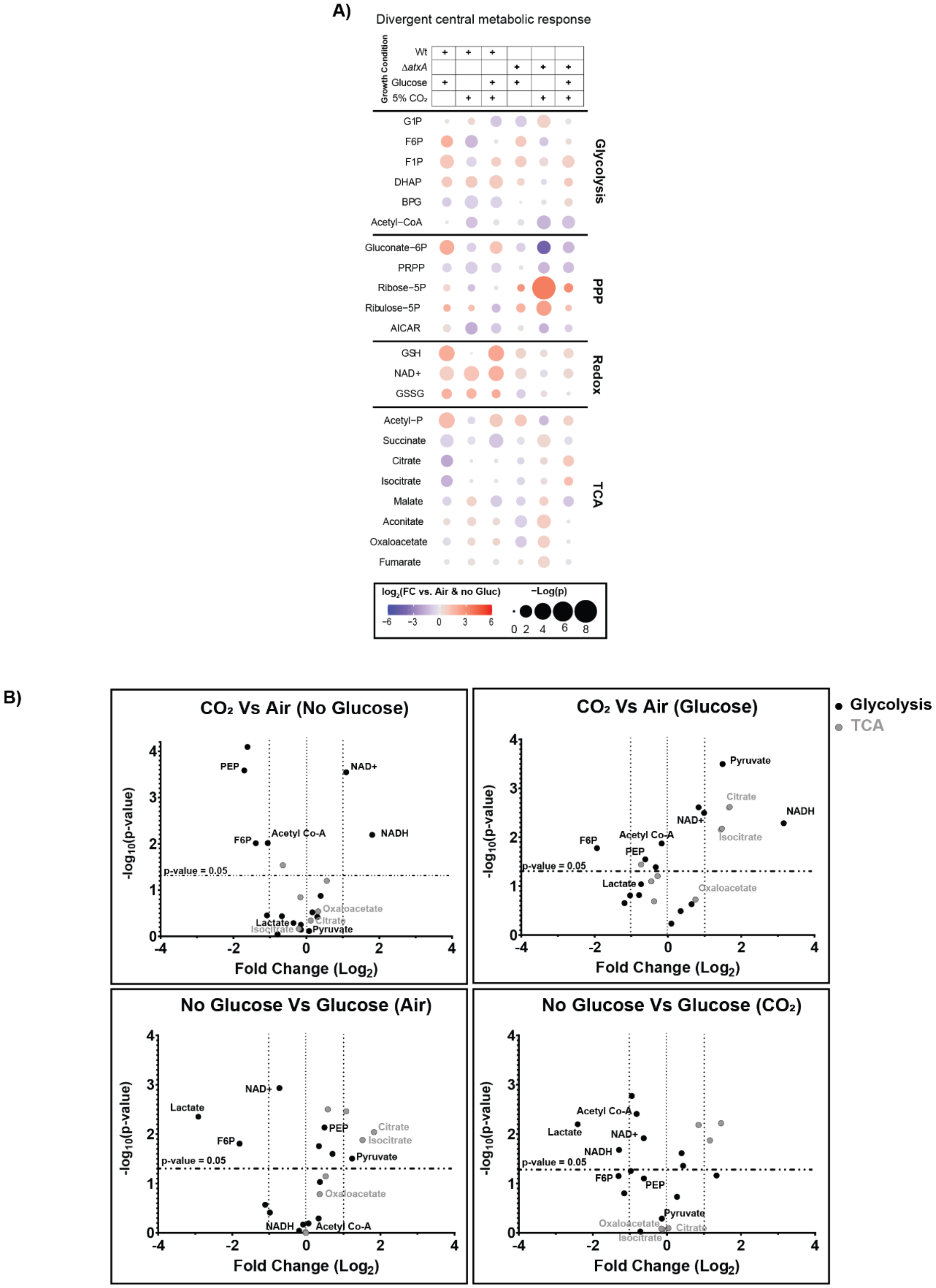

A) Bubble plot showing the relative abundance of divergent carbon metabolism metabolites significantly dysregulated under different growth conditions in *B. anthracis* Wt and *ΔatxA*. The

bioRxiv preprint doi: https://doi.org/10.1101/2025.10.02.679957; this version posted October 7, 2025. The copyright holder for this preprint (which was not certified by peer review) is the author/funder. This article is a US Government work. It is not subject to copyright under 17 USC 105 and is also made available for use under a CC0 license.

color gradient (red to blue) indicates fold change in metabolite levels compared to air and no-glucose conditions. Bubble size represents the p-value as -log(p).

B) Scatter plot of selected glycolysis (black dots) and TCA (grey dots) intermediates comparing several growth conditions. Growth conditions for each comparison are mentioned at the top of each graph. The comparisons are made as following: Variable-1 vs Variable-2 (constant). The x-axis indicates fold change in metabolite levels and y-axis represents the p-value as -log(p).

bioRxiv preprint doi: https://doi.org/10.1101/2025.10.02.679957; this version posted October 7, 2025. The copyright holder for this preprint (which was not certified by peer review) is the author/funder. This article is a US Government work. It is not subject to copyright under 17 USC 105 and is also made available for use under a CC0 license.

Supplementary figure 3.

A)

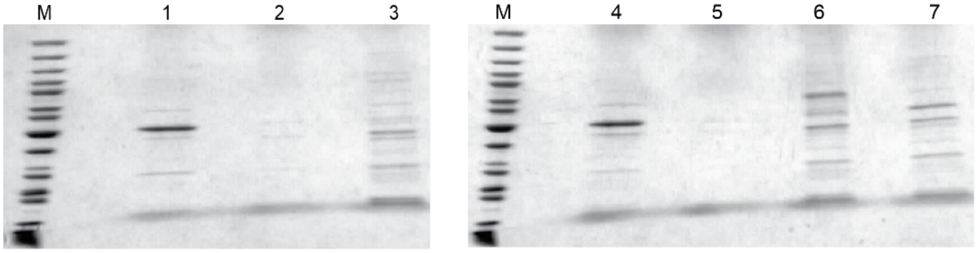

	Label	Name	Description
Air	M	Protein ladder	
	1	Wt :: pAMY-AtxA	Wild type over-expressing AtxA
	2	Δ*atxA* :: pAMY1	AtxA null mutant with empty vector **(Negative)**
	3	Δ*atxA* :: pAMY-AtxA	AtxA null mutant over-expressing AtxA **(Pulled down)**
5% CO_2	4	Wt :: pAMY-AtxA	Wild type over-expressing AtxA
	5	Δ*atxA* :: pAMY1	AtxA null mutant with empty vector **(Negative)**
	6	Δ*atxA* :: pAMY-AtxA	AtxA null mutant over-expressing AtxA **(Pulled down)**
	7	Δ*atxA* :: pAMY1 spiked AtxA-H199D	AtxA null mutant with empty vector and spiked with AtxA-H199D

B)

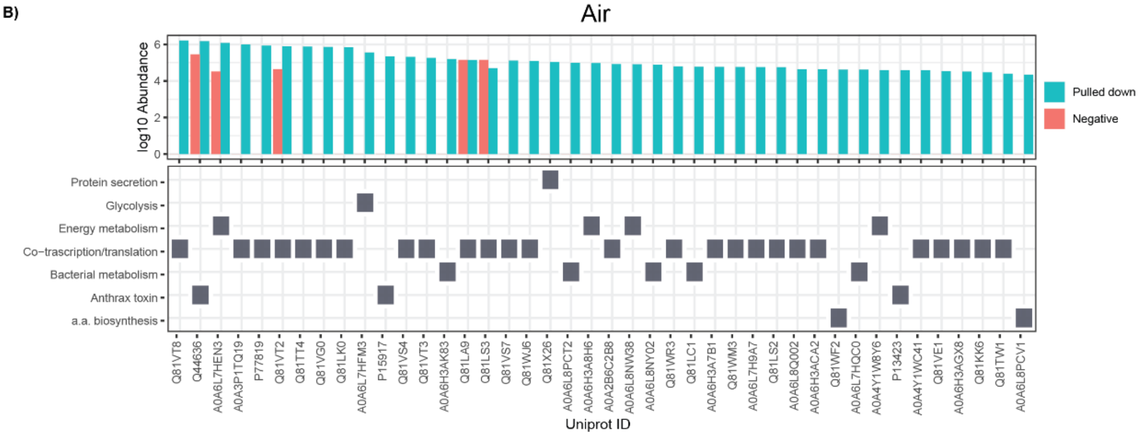

C)

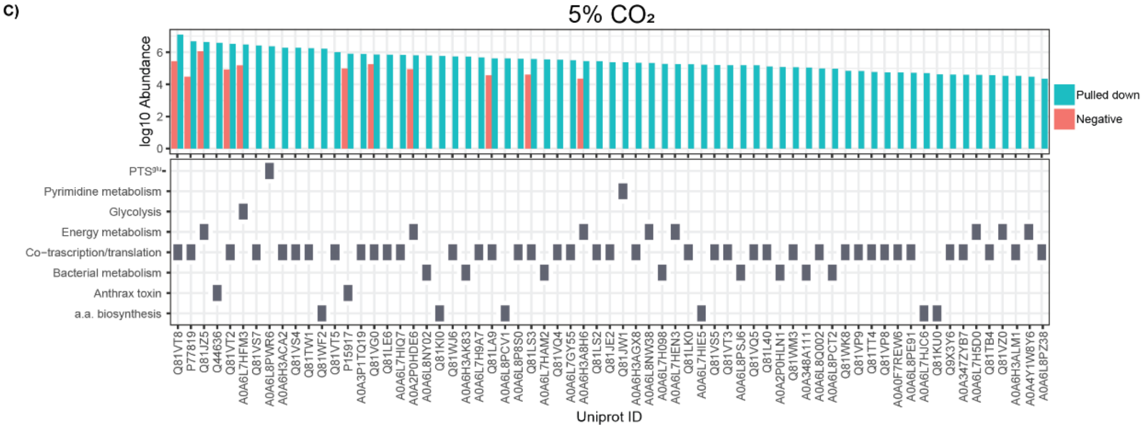

A) Coomassie-stained polyacrylamide gel of the elutes from co-affinity samples. Lanes are marked from 1-7 for the list of strains and growth conditions used in the table below. M = Protein molecular ladder.

B,C) Bar graphs showing the absolute abundance (y-axis) of peptides for respective proteins (Uniprot IDs, x-axis) identified from co-affinity purification samples. Cyan bars indicate abundance in the pulled-down sample, while red bars indicate background or non-specific proteins. Each

bioRxiv preprint doi: https://doi.org/10.1101/2025.10.02.679957; this version posted October 7, 2025. The copyright holder for this preprint (which was not certified by peer review) is the author/funder. This article is a US Government work. It is not subject to copyright under 17 USC 105 and is also made available for use under a CC0 license.

protein is classified based on its molecular function in the table below. B) AtxA interactions identified from bacteria grown in air. C) AtxA interactions identified from bacteria grown in 5% CO_2. Glucose was present in both conditions.

bioRxiv preprint doi: https://doi.org/10.1101/2025.10.02.679957; this version posted October 7, 2025. The copyright holder for this preprint (which was not certified by peer review) is the author/funder. This article is a US Government work. It is not subject to copyright under 17 USC 105 and is also made available for use under a CC0 license.

Supplementary figure 4.

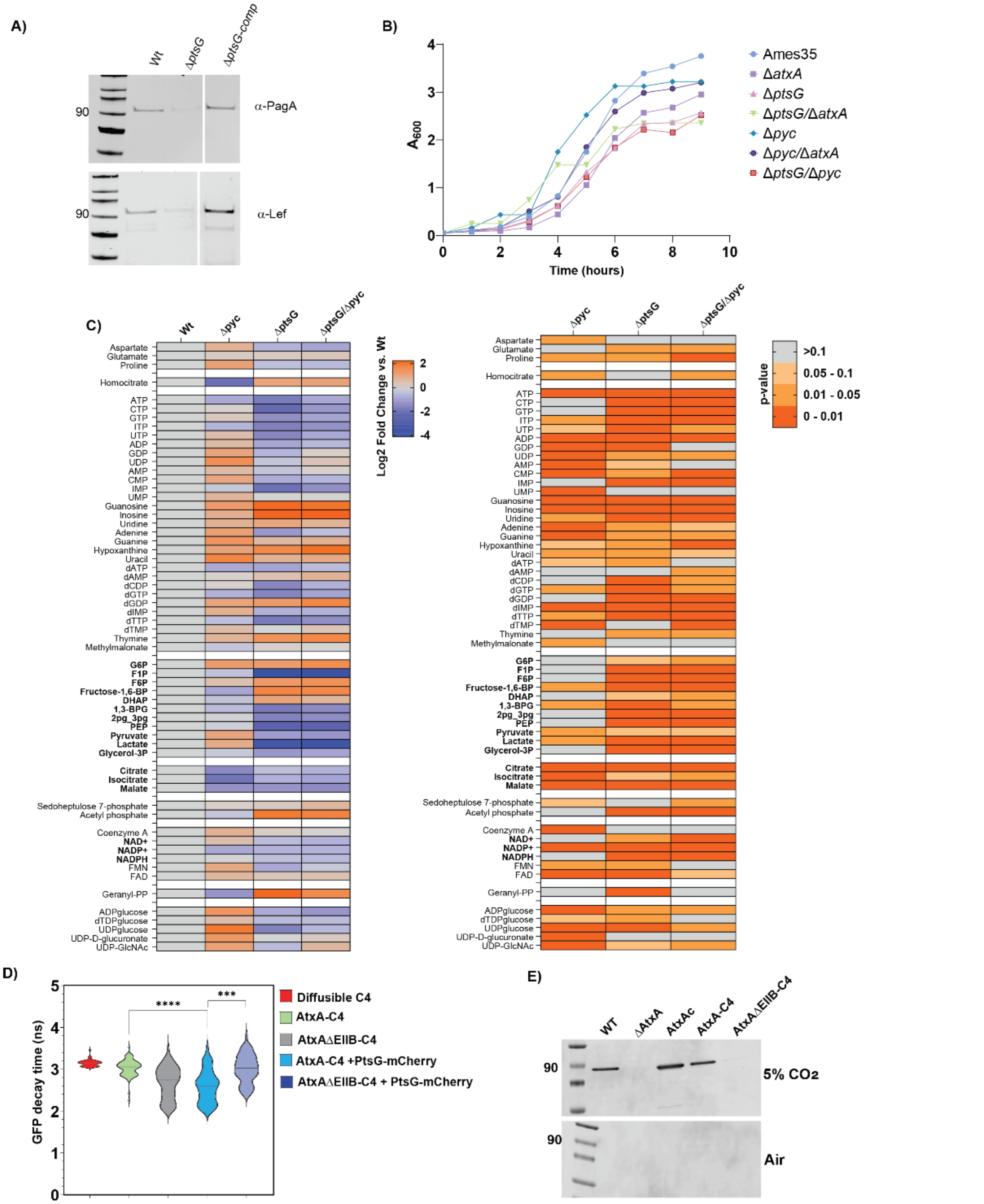

A) Immunoblotting for anthrax toxin components (PagA and Lef) in *B. anthracis* WT, Δ*ptsG*, and its complemented strain (Δ*ptsG*-Comp). Supernatant volumes were normalized against total cellular protein for comparative analysis. Representative graph of n=2 experiments.

bioRxiv preprint doi: https://doi.org/10.1101/2025.10.02.679957; this version posted October 7, 2025. The copyright holder for this preprint (which was not certified by peer review) is the author/funder. This article is a US Government work. It is not subject to copyright under 17 USC 105 and is also made available for use under a CC0 license.

B) Growth kinetics of all *B. anthracis* strains under toxin-producing conditions (glucose + 5% CO_2). Each data point represents the average of three replicates. Representative image of n=2 experiments.

C) Heatmap showing the relative abundance of significantly altered metabolites in Δ*ptsG*, Δ*pyc*, and Δ*ptsG*/Δ*pyc* mutants compared to their respective abundance in *B. anthracis* WT strain grown under toxin-producing conditions (glucose + 5% CO_2). The left heatmap shows the log2 fold change in abundance as a color gradient from blue (low abundance) to red (high abundance). The right heatmap corresponds to the p-value of each data point on the left heatmap, represented as a color gradient from grey ($p > 0.1$, insignificant) to orange ($p = 0$-0.01, highly significant).

D) Representative FRET-FLIM based interactions in strains expressing different FRET partners. The violin/distribution plot represents the median GFP decay profile (in nanoseconds, ns) GFP variant C4 in the respective strains. The graph represents n=2 experiments, * indicates p-value.

E) Immunoblotting for PagA showing effects of loss of the EIIB domain of AtxA (AtxA-ΔEIIB-C4) on anthrax toxin expression. Having AtxA variants expressed in the background of *B. anthracis ΔatxA* strains, with plasmid pAMY expressing the respective AtxA variants. Representative image of n=3 experiments.

bioRxiv preprint doi: https://doi.org/10.1101/2025.10.02.679957; this version posted October 7, 2025. The copyright holder for this preprint (which was not certified by peer review) is the author/funder. This article is a US Government work. It is not subject to copyright under 17 USC 105 and is also made available for use under a CC0 license.

Supplementary figure 5.

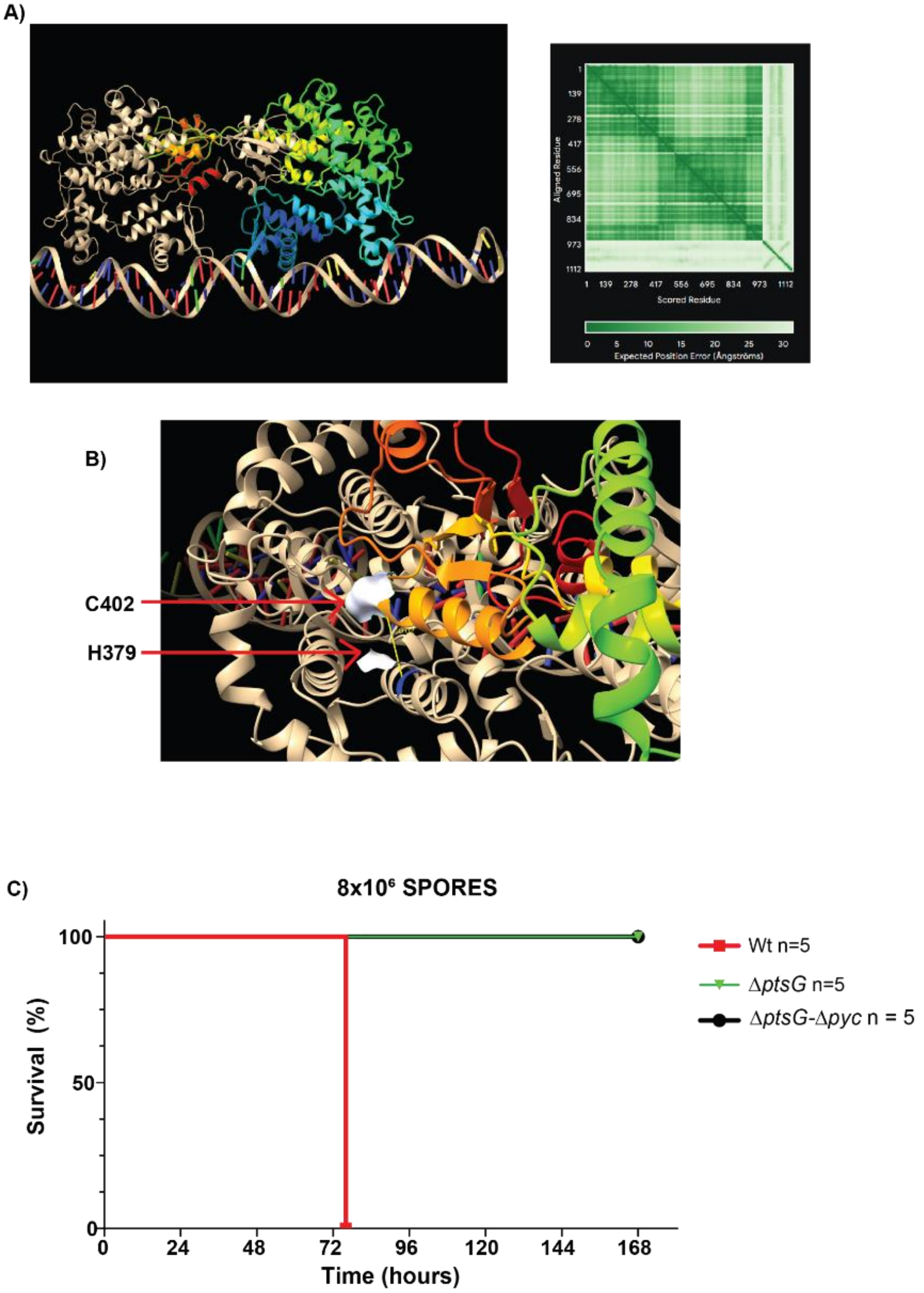

A, B) Alpha-fold predicted structure of WT AtxA dimer with the *pagA* promoter carrying AtxA-binding elements. The two units of the AtxA dimer are shown in rainbow and golden colors. B) Zoomed image of the interaction between C402 of one unit (rainbow-colored) and H379 of the other unit (golden-colored). The electrostatic interaction zones of both residues are shown as white clouds around them.

bioRxiv preprint doi: https://doi.org/10.1101/2025.10.02.679957; this version posted October 7, 2025. The copyright holder for this preprint (which was not certified by peer review) is the author/funder. This article is a US Government work. It is not subject to copyright under 17 USC 105 and is also made available for use under a CC0 license.

C) Virulence of parent and PTS^{glu} and Pyc mutants. A. Survival curves of mice infected sub-cuteneously (s.c.) with spores *B. anthracis* are shown. C57BL/6J mice were injected s.c. with $8X10^6$ CFU of the Wt (red line; n = 5), Δ*ptsG* (green ; n=5) , and Δ*ptsG/* Δ*pyc* (black ; n=5).
